# Effects of Aging on Mode I Fatigue Crack Growth Characterization of Double Cantilever Beam Specimens with Thick Adhesive Bondline for Marine Applications

**DOI:** 10.3390/ma18143286

**Published:** 2025-07-11

**Authors:** Rahul Iyer Kumar, Wim De Waele

**Affiliations:** 1 Soete Laboratory, Department of Electromechanical, Systems and Metal Engineering, Ghent University, 9000 Ghent, Belgium; rahul.iyerkumar@ugent.be; 2 FlandersMake@UGent–Corelab MIRO, 9000 Ghent, Belgium

**Keywords:** adhesive joint, fatigue crack growth, double cantilever beam, marine and offshore structures, structural adhesive

## Abstract

The use of adhesive joints in naval applications requires a thorough understanding of their fatigue performance. This paper reports on the fatigue experiments performed on double cantilever beam specimens with thick adhesive bondline manufactured under shipyard conditions. The specimens have an initial crack at the steel–adhesive interface and are tested in unaged, salt-spray-aged and immersion-aged conditions to determine the interface mode I fatigue properties. The strain energy release rate is calculated using the Kanninen–Penado model, and the fatigue crack growth curve is determined using a power law model. The crack growth rate slope for salt-spray-aged specimens is 16.5% lower than for unaged specimens, while that for immersion-aged specimens is 66.1% lower and is shown to be significantly different. The fracture surfaces are analyzed to identify the failure mechanisms and the influence of the aging process on the interface properties. Since the specimens are manufactured under shipyard conditions, the presence of voids and discontinuities in the adhesive bondline is observed and as a result leads to scatter. Hence, Bayesian linear regression is performed in addition to the ordinary least squares regression to account for the scatter and provide a distribution of plausible values for the power law coefficients. The results highlight the impact of aging on the fatigue property, underscoring the importance of considering environmental effects in the qualification of such joints for marine applications.

## 1. Introduction

Recently, there has been a greater emphasis on the use of fiber-reinforced polymer composites in ships by naval engineers [1,2]. This is driven by the need to improve performance, reduce ownership cost and minimize the environmental impact of ships [3]. Additionally, the use of composite materials in naval applications improves stealth capabilities, which is vital in modern warfare [4,5]. However, the incorporation of dissimilar materials necessitates the use of adhesive bonding over mechanical fastening techniques, such as bolts and rivets. This preference arises from the superior strength-to-weight ratio achievable and the reduction in stress concentration zones along the joint [6]. Since building docks are often located near the coast, banks of rivers or quays, the entire manufacturing process is exposed to the elements [7,8]. This leads to challenges in the surface preparation of the adherends and maintaining close tolerances between the adherends, which give rise to bondline thickness in the *millimeter* range [9,10,11]. During the service life of the ship, these adhesive bonds are subjected to a combination of dynamic mechanical loading, corrosive saline environment and temperature variations; this necessitates the need for a thorough understanding of the joints’ long-term performance under representative conditions. Standards for determining the fatigue properties of adhesively bonded joints typically follow strength-based approaches [12,13]. However, there is no specific standard that is based on the fracture mechanics approach [14].

Fracture mechanics-based standards for metals and fiber-reinforced polymer (FRP) are often repurposed to evaluate the fatigue crack growth (FCG) relationship of adhesive joints [15,16,17]. To this end, researchers performed experiments on double cantilever beam (DCB) specimens to determine the relationship between the crack growth rate ($\frac{da}{dN}$) and a fracture mechanics parameter such as the strain energy release rate (*G*) [18,19,20,21,22,23,24]. This is usually presented as a log–log plot of $\frac{da}{dN}$ and $G_{\max}$ which typically yields a sigmoidal curve where the middle linear region is the stable crack growth region and can be approximated by a simple power law given by Equation (1), where *C* and *m* are material constants. The exponent *m*, analogous to the slope of the linear region, is a measure of the material’s load sensitivity of the crack propagation rate:

(1) $$\frac{da}{dN}=C{\left(G_{\max}\right)}^{m}$$

Mall and Ramamurthy experimented to evaluate the effect of the bondline thickness (0.102 mm, 0.254 mm and 0.508 mm) of DCB specimens with composite adherends bonded with epoxy adhesive [18]. They calculated the strain energy release rate under mode I loading ($G_{I}$) and the compliance (C) based on linear beam theory and observed that at lower growth rates, the fatigue resistance is independent of the bondline thickness. However, at higher growth rates, the fatigue resistance of the 0.508 mm thick bondline specimen increased in comparison to the other two. Azari et al. studied the effect of adherend thickness on the FCG behavior of epoxy adhesive using symmetric and asymmetric DCB specimens [25]. The adhesive bondline thickness in their study is maintained constant for all specimens at 0.38 mm and an analytical beam on elastic foundation model developed by Fernlund and Spelt is used to calculate the strain energy release rate [26]. They concluded that the FCG and adherend thickness are directly proportional. Rocha et al., on the other hand, performed experiments on three different adhesive systems (epoxy based, acrylic based, and rubber based) with bondline thicknesses of 0.30 mm and 0.05 mm and used the compliance-based beam method (CBBM) to calculate the strain energy release rate (SERR) under mode I, mode II, and mixed mode loading [27]. The authors discussed the crack growth and failure mechanism, but unfortunately no valid comparison was established owing to the different thicknesses for each adhesive system. Xu et al. evaluated the effect of testing frequency on the FCG rate of DCB specimens with bondline thickness of 0.2 mm and 1.0 mm and they observed that the FCG rate increases with decreasing frequency [28]. Finite element analysis was used to calculate G and J-integral in this research. Abou-Hamda et al. utilized the formulations of Yamada to determine the J-integral of DCB specimens with aluminum adherends bonded together using epoxy-based adhesive and having bondline thicknesses of 0.3 mm, 0.8 mm and 1.0 mm, and concluded that the thicker bondline showcases higher fatigue crack resistance [29,30]. The recent research by Fan et al. looked into the effect of the voids and displacement ratio ($R_{d}$-ratio) on the FCG of epoxy-based structural adhesive with a thick bondline (10.0 mm) and made use of the corrected beam theory to calculate the SERR. They showed that the FCG is $R_{d}$-ratio independent in specimens with voids, indicating that the voids dominate the fatigue behavior [31].

The aforementioned studies show varied ways to calculate the SERR values (G) under mode I loading condition; however, the adhesives used are largely limited to epoxy-based adhesives. Limited research has been conducted on acrylic-based structural adhesives. Pirondi and Nicoletto performed experiments on DCB specimens bonded with methacrylate-based structural adhesive with a bondline thickness of 0.3 mm to evaluate the influence of testing frequency and R-ratio on the FCG response [22]. They used the solution developed by Krenk [32], based on an elastic foundation, which accounts for the elastic behavior of the adhesive and the bondline thickness to determine the SERR, and determined that the R-ratio has a higher influence on the FCG than the testing frequency. Kim et al. studied the behavior of acrylic adhesive with different adherend materials and found that FCG is independent of the adherends [33]. Imanaka et al. studied the effect of variation in aluminum adherend thickness in a CFRP-aluminum DCB specimen and found that the threshold value of SERR $\left(\Delta G_{\mathrm{th}}\right)$ increased with an increase in the adherend thickness [34]. Sekiguchi and Sato also performed experiments on acrylic structural adhesives with varying bondline thickness (0.15 mm to 0.82 mm) and observed that the fatigue crack resistance $\left(\Delta G\right)$ was greater for thicker bondlines at a low number of cycles $\left(1\times 10^{4}\right)$ and no influence of thickness at higher numbers of cycles [24]. It is noteworthy that the research discussed above is limited to thin bondlines below 1.0 mm, except for the study by Fan et al. [31]. To the best of the authors’ knowledge, very limited research has been conducted to evaluate the fatigue properties of acrylic-based structural adhesives with a thick bondline. To that effect, the current research investigates the mode I steel–adhesive interface property of an adhesively bonded DCB specimen with thick bondline under fatigue loading. Additionally, the research also investigates the influence of aging on the interface properties. Since this research is part of the multi-year, multi-partner European Union project QUALIFY, the aging methodologies employed—salt-spray aging and immersion aging in a saltwater bath at elevated temperature—are kept consistent to compare the results within the project [35].

## 2. Materials and Methods

### 2.1. Specimen Configuration and Preparation

**Figure 1:**
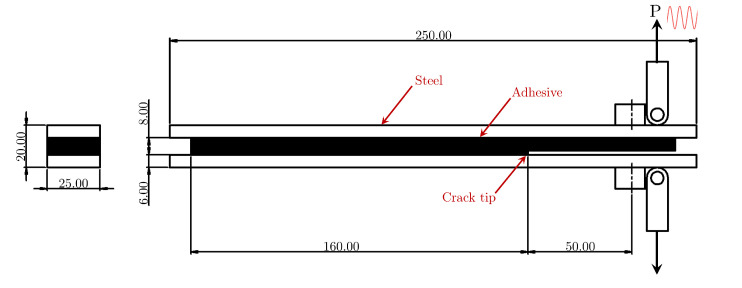
A schematic diagram of the DCB specimen and the loading pins, indicating the initial crack tip location at the interface of steel and adhesive. The dimensions shown are in millimeters. The width of the DCB specimen is 25 mm.

Experiments are performed on DCB specimens (Figure 1) that consist of 6 mm thick steel adherends bonded together with a structural adhesive having a nominal thickness of 8 mm, with the initial crack located at the interface of the adhesive and the adherend.

The DCB specimens are manufactured under shipyard conditions by the QUALIFY project partner Damen Naval, where two AH36 steel plates (250 mm × 250 mm) of thickness 6 mm are bonded using a two-component methyl-methacrylate (MMA) adhesive. A nominal bondline thickness of 8 mm is achieved by using spacers. The steel adherend surfaces are prepared by grit blasting up to SA 2.5 standard [36], followed by degreasing and cleaning with isopropyl alcohol. A polytetrafluoroethylene (PTFE) sheet is placed on one of the steel–adhesive interfaces before bonding to create a discontinuity in the bondline, representing the initial crack. The bonded plates are cured at room temperature for 24 h and water-jet cut to the required dimensions. An initial crack length ($a_{0}$) of 50 mm between the crack tip and the loading pin is maintained for all specimens.

**Figure 2:**
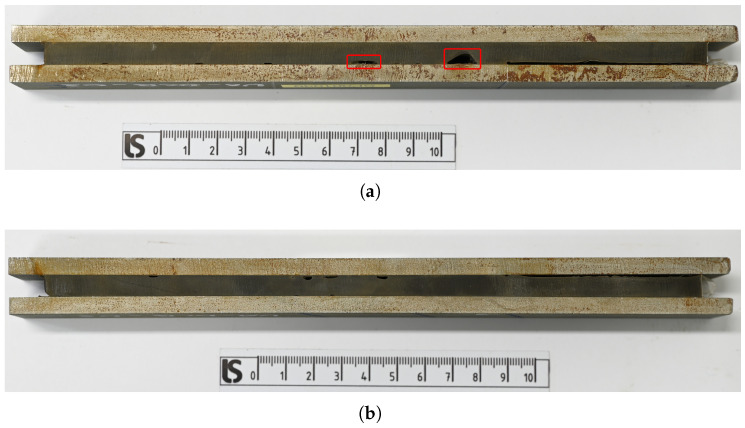
Images of the DCB specimens received after water jet cutting, showing variations in the amount and size of imperfections visible at the side face of the specimen. (**a**) Specimen UA-DCB-03 shows larger defects compared to (**b**) specimen UA-DCB-05.

Figure 2 shows the images of two DCB specimens as received after water jet cutting, highlighting variations in the quantity and size of imperfections visible at the specimen surface. Specimens UA-DCB-03 exhibit larger imperfections (marked with red boxes) which are located closer to the initial crack position compared to UA-DCB-05. The presence of these defects is crucial, as they replicate the real-world manufacturing conditions found in shipyards. Testing these specimens ensures that the results are representative of practical scenarios but conservative. In contrast, testing lab-manufactured, defect-free specimens could potentially skew the results by failing to account for the inherent variability and imperfections encountered in actual applications.

### 2.2. Aging Procedure

A total of 12 specimens are manufactured, with 6 specimens tested in as-manufactured condition, hereinafter referred to as the unaged condition, and the remaining 6 specimens tested after aging. The specimens are aged according to the ASTM B117 standard [16]; the samples are placed in a salt-spray chamber with 5% salinity, 35 °C and 50% humidity for a period of 6 weeks. Of the six salt-spray-aged specimens, three specimens are additionally aged by immersion in a saltwater bath containing 3.5% NaCl maintained at 50 °C for 1 week. The additional aging is a harsher condition than what the joint is subjected to on an actual ship since the joint is never under the sea level. Furthermore, this facilitates comparison with other tests performed under the QUALIFY project umbrella and enables to observe the effect of immersion aging on the adhesive behavior [37].

### 2.3. Experimental Procedure

As mentioned in Section 1, there are no standards that describe an experimental procedure to determine the mode I fatigue properties of adhesively bonded joints with metal adherend and thick adhesive bondline. The experimental procedure adopted in this work is partly based on the ASTM D6115-97 standard, which describes the test procedure for determining the mode I fatigue delamination growth onset in composites. According to the standard, the DCB specimen is subjected to cyclic loading under displacement control at a constant frequency. The maximum cyclic displacement $\left(\delta_{max}\right)$ to be applied during the test is set according to Equation (2), where ${\left(\delta_{\mathrm{crit}}\right)}_{\mathrm{avg}}$ is the average critical displacement for delamination growth under quasi-static load:

(2) $$\frac{\delta_{\max}^{2}}{{\left(\delta_{\mathrm{crit}}\right)}_{\mathrm{avg}}^{2}}=0.5$$

The standard recommends the fatigue test to be performed under displacement control with displacement ratio in the range $0\leq \frac{\delta_{min}}{\delta_{max}}<1$ and the testing frequency to be between 1 and 10 Hz. As per the standard, the fatigue test stops upon reaching either a predefined compliance or a predetermined number of cycles.

The current research modifies the standard’s recommendation by performing the fatigue test under load control. A block-loading scheme similar to the one used in previous research is implemented where the load applied to the specimen is increased after a certain number of cycles [38]. This scheme has the flexibility of starting at a low load close to the threshold value of strain energy release rate and also having sufficient data points in the stable crack growth region. One drawback of a load-controlled experiment is that care must be given in choosing the loads for the first loading block; a high starting load can lead to rapid crack growth, and a low load can lead to no crack growth even after millions of cycles.

Analogous to the procedure recommended in the standard, the average critical load ${\left(F_{\mathrm{crit}}\right)}_{\mathrm{avg}}$ at which the crack starts to propagate during a quasi-static test is determined and used as reference for the maximum load $\left(F_{\max}\right)$ applied during the fatigue test. To start with, a value $F_{\max}=30\%{\left(F_{\mathrm{crit}}\right)}_{\mathrm{avg}}$ is applied, and the load is increased by 5% ${\left(F_{\mathrm{crit}}\right)}_{\mathrm{avg}}$ at the end of each loading block. The value of ${\left(\delta_{\mathrm{crit}}\right)}_{\mathrm{avg}}$ and corresponding ${\left(F_{\mathrm{crit}}\right)}_{\mathrm{avg}}$ are obtained from quasi-static tests performed by QUALIFY project partners on identical DCB specimens [39,40].

The fatigue experiments are performed on a servo-hydraulic testing machine with a load cell of 5 kN capacity. A load ratio of R = 0.1 and a testing frequency of 4 Hz are maintained during the fatigue test. A dual-camera setup is employed to monitor the progression of the interface crack at the front and rear sides of the specimen. These cameras capture a set of 25 images every 5000 cycles throughout the test simultaneously. The data acquisition system also records the load and actuator displacement at the moment each image is captured. The DIC technique was applied to a limited number of specimens to assess the strain field in the adhesive. However, its use was restricted because the black speckles interfered with the accurate tracking of the crack front.

### 2.4. Data Reduction

The mode-I strain energy release rate $\left(G_{I}\right)$ for bonded joints is determined by the Irwin–Kies Equation (3) where *P* is the applied load, *B* the specimen width, *C* the compliance, and *a* the crack length:

(3) $$G_{I}=\frac{P^{2}}{2B}\frac{dC}{da}$$

**Figure 3:**
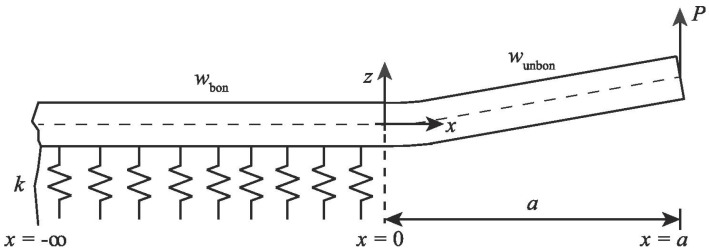
A schematic representation of a DCB specimen modeled according to the Kanninen–Penado model [44]. Reproduced with permission from Lopes Fernandes, R et al., Engineering Fracture Mechanics; published by Elsevier, 2019.

Various methods exist to determine the compliance *C* [14]. ASTM D6115-97 recommends the use of the modified beam theory (MBT), the compliance calibration method (CCM), or the modified compliance calibration method (MCCM) applied to a fiber-reinforced polymer composite specimen. These methods, however, neither account for the rotation of the beam at the crack tip nor for the flexibility of the adhesive layer, especially in thicker bondlines. In the current research, the beam on elastic foundation model is used, specifically the model proposed by Kanninen applied to a homogeneous DCB [41] and extended to a bonded DCB by Penado [42]. Researchers have used the Kanninen–Penado model to evaluate the SERR of DCB specimens under static loading [43,44]. Additionally, the models’ accuracy under fatigue loading has also been compared, and it was found to perform well for different metallic substrates and loading conditions [14,45]. The model considers the DCB arm to be an Euler–Bernoulli beam, which is free at one end and supported by an elastic foundation at the other end representing the unbonded and bonded regions, respectively. The model considers the DCB to be symmetric with respect to a plane coinciding with the half-thickness of the adhesive as shown in Figure 3.

According to the Kanninen–Penado model, the SERR under the mode I loading condition is given by Equation (4), where *P* is the load applied, *a* the crack length, *E* the elastic modulus of the adherend, and *I* the moment of inertia of the beam. The parameter $\lambda^{-1}$ is the process zone length which is interpreted as the distance from the crack tip over which the positive peel stress is distributed, which depends on the stiffness of the foundation *k*, which in turn is a function of the elastic modulus of the adhesive $E_{ad}$, the beam width *B*, and half-bondline thickness *t* [43]:

(4) $$G_{I}=\frac{P^{2}}{BEI\lambda^{2}}\left(\lambda^{2}a^{2}+2\lambda a+1\right)\mathrm{where}\;\;\;\lambda =\sqrt[{4}]{\frac{k}{4EI}}$$

The model was originally developed for a DCB specimen with a crack positioned at the mid-plane of the adhesive layer. However, in this study, the crack is intentionally placed at the steel–adhesive interface, introducing asymmetry both in the specimen and, more critically, at the crack tip. To validate the model’s applicability to the current specimen configuration, the authors conducted a preliminary experimental study in which the process zone length from the crack tip and the loading condition at the crack tip was measured using DIC data. The results of the study confirmed that the process zone length calculated by the Kanninen–Penado model and the one measured by DIC are in good agreement. It was also shown that during loading, mode I remains the dominant loading condition at the crack tip [46].

### 2.5. Crack Length Measurement and Fracture Surface Analysis

**Figure 4:**
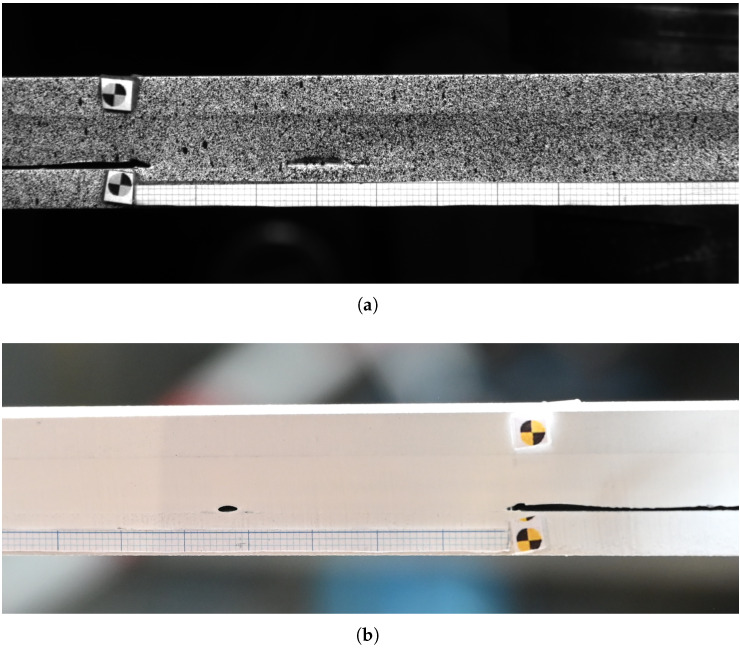
Cropped images of the DCB specimen captured during the experiment by (**a**) the DIC cameras placed in front of the specimen and (**b**) a digital camera placed at the back of the specimen.

Figure 4a,b show the cropped images of both the front of a DCB specimen captured by the DIC cameras and the back captured by a digital camera, respectively. As mentioned previously (Section 2.3), both cameras capture a set of 25 images every 5000 cycles, and the crack length is measured from the image that corresponds to the maximum load within this set. A millimeter paper is attached to the steel adherend on both sides of the specimen to help measure the crack length during the experiment. This crack length is measured using the image processing software Fiji v2.16.0 [47]. VIC-3D 8 software from Correlated Solutions is used to post-process the DIC images and to evaluate the strain distribution at the surface of the specimen.

**Figure 5:**
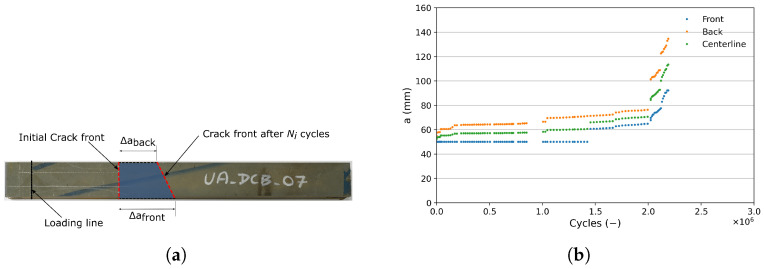
(**a**) An exaggerated representation of the crack length at the front and back of the DCB specimen as viewed from the top. This situation can arise due to the presence of pores and discontinuities. The shaded region represents the disbond area after $N_{i}$ cycles. (**b**) Plot of the crack length measured at the front and back of the DCB specimen UA-DCB-04. The crack length at the center-line of the specimen is calculated according to Equation (5), which is eventually used for the SERR calculation.

Due to the voids and discontinuity in bonding within the DCB specimens, the crack length measured on the two faces of the DCB specimens at a particular cycle may not be the same. Figure 5a shows an exaggerated top-view schematic representation of the crack growth at the front $\left(\Delta a_{\mathrm{front}}\right)$ and back $\left(\Delta a_{\mathrm{back}}\right)$ of the DCB specimen. The red dotted lines represent the crack front at the start of the experiment and after $N_{i}$ cycles, assuming a straight-line crack progression. Due to this inconsistency, the crack growth at the center of the specimen $\left(\Delta a\right)$ is considered for SERR calculation and is given by Equation (5):

(5) $$\Delta a=\frac{1}{2}\left|\Delta a_{\mathrm{front}}-\Delta a_{\mathrm{back}}\right|+\min\left(\Delta a_{\mathrm{front}},\Delta a_{\mathrm{back}}\right)$$

In order to evaluate the features of the fracture surface, the specimens are scanned using a Keyence VR-5200 3D surface profilometer (Keyence International NV/SA, Mechelen, Belgium), which captures optical images of the fracture surface as well as the surface profile of the fracture surface. These scans also allow to investigate the quality of bonding, the presence of voids, and the mode of failure that occurred during the test.

### 2.6. Statistical Analysis

The coefficients of the power law expressing the FCGR fatigue crack growth rate (Equation (1)) for unaged, salt-spray-aged, and immersion-aged specimens are determined by performing ordinary least squares (OLS) regression on the experimental data. In order to statistically compare the various treatments (unaged, salt-spray aging, and immersion-aging) of the specimens, a variation of the OLS regression model known as moderated multiple regression (MMR) model is employed. This model makes use of multiplicative interaction terms in the regression model, and the corresponding coefficients of the interactions are compared using analysis of covariance (ANCOVA) test [48,49]. It tests for the null hypothesis $H_{0}$ that the slope of the fitted linear regression of unaged specimens and the different aged specimens belong to the same population, and an alternate hypothesis $H_{1}$ that the slopes are different with a significance level of α=0.05. A brief description of the MMR model is given in Section 2.6.1:

$$\mathrm{Unaged}\;\mathrm{and}\;\mathrm{salt-spray-aged}\;\mathrm{specimens}\left\{\begin{array}{cc} H_{0}: & m_{\mathrm{unaged}}=m_{\mathrm{saltspray}}\\ H_{1}: & m_{\mathrm{unaged}}\neq m_{\mathrm{saltspray}} \end{array}\right.$$

$$\mathrm{Unaged}\;\mathrm{and}\;\mathrm{immersion-aged}\;\mathrm{specimens}\left\{\begin{array}{cc} H_{0}: & m_{\mathrm{unaged}}=m_{\mathrm{immersion}}\\ H_{1}: & m_{\mathrm{unaged}}\neq m_{\mathrm{immersion}} \end{array}\right.$$

Additionally, the power law coefficient parameters are also quantified using Bayesian linear regression and No U-Turn Sampler (NUTS). Although Bayesian methods have been used to estimate model parameters and fatigue lifetime under complex loading conditions [50,51,52], in this research it is implemented to estimate the distribution of the FCGR parameters, taking into account the uncertainty in the data.

#### 2.6.1. Moderated Multiple Regression

To understand the influence of aging on the interfacial crack growth rate, it is necessary to statistically quantify if the datasets belong to a single population or not. Hence, a multiplicative interaction term is included in the general regression model to compare the slopes of the log–log linear regression curves relating $G_{\max}$ and $\frac{da}{dN}$ for categorical factors containing j=3 levels, i.e., unaged, salt-spray-aged, and immersion-aged specimens. The general equation for the moderated multiple regression model with interaction is

(6) $$\begin{array}{cc} Y_{i}= & \beta_{0}+\beta_{1}\left(G_{\max,i}\right)+\beta_{2}I_{\mathrm{saltspray},i}+\beta_{3}I_{\mathrm{saltspray},i}\left(G_{\max,i}\right)\\ & \;\;\;\;\;\;\;\;\;\;\;\;\;\;\;+\beta_{4}I_{\mathrm{immersion},i}+\beta_{5}I_{\mathrm{immersion},i}\left(G_{\max,i}\right) \end{array}$$

where $I_{\mathrm{saltspray},i}$ and $I_{\mathrm{immersion},i}$ are indicator variables for the categorical factors. The terms containing only the indicator variables allow intercepts to vary among the level of categorical factors, whereas the terms containing the cross-product of indicator variables and $G_{\max}$ allow the slopes to vary. For observations in the category group unaged specimen, the indicator variables take the value 0, i.e., $I_{\mathrm{saltspray},i}=0$ and $I_{\mathrm{immersion},i}=0$ in Equation (6). This gives

(7) $$Y_{i}=\beta_{0}+\beta_{1}\left(G_{\max,i}\right)$$

which has unaged specimen’s intercept $\beta_{0}$ and quantitative slope $\beta_{1}$. Similarly for observations in the salt-spray-aged specimen group, the indicator variables take the value $I_{\mathrm{saltspray},i}=1$ and $I_{\mathrm{immersion},i}=0$, and for observations in the immersion-aged specimen group, the values are $I_{\mathrm{saltspray},i}=0$ and $I_{\mathrm{immersion},i}=1$, which simplifies to Equations (8) and (9), respectively:

(8) $$Y_{i}=\left(\beta_{0}+\beta_{2}\right)+\left(\beta_{1}+\beta_{3}\right)\left(G_{\max,i}\right)$$

(9) $$Y_{i}=\left(\beta_{0}+\beta_{4}\right)+\left(\beta_{1}+\beta_{5}\right)\left(G_{\max,i}\right)$$

Linearizing the power law (Equation (1)) by applying a log–log transformation and comparing it with Equation (7), it is evident that the $\beta_{0}$ and $\beta_{1}$ are analogous to the logC and *m*, which are the intercept and slope of the regression line, respectively, and the dependent variable $Y_{i}$ is analogous to $\log{\frac{da}{dN}}_{i}$. The terms $\beta_{3}$ and $\beta_{5}$ in Equations (8) and (9) represent the interaction term which modifies the slope coefficient due to the effect of salt-spray and immersion aging, respectively. Hence, the null hypothesis and the alternate hypothesis can be modified in terms of β as

$$\mathrm{Unaged}\;\mathrm{and}\;\mathrm{salt-spray-aged}\;\mathrm{specimens}\left\{\begin{array}{cc} H_{0}: & \beta_{3}=0\\ H_{1}: & \beta_{3}\neq 0 \end{array}\right.$$

$$\mathrm{Unaged}\;\mathrm{and}\;\mathrm{immersion-aged}\;\mathrm{specimens}\left\{\begin{array}{cc} H_{0}: & \beta_{5}=0\\ H_{1}: & \beta_{5}\neq 0 \end{array}\right.$$

## 3. Results and Discussions

**Figure 6:**
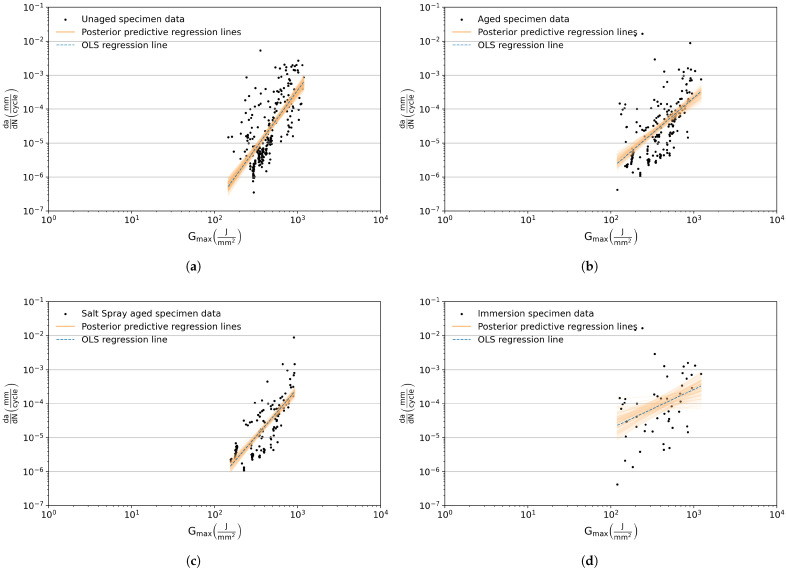
FCG results of the (**a**) unaged and (**b**) aged DCB specimens. An additional distinction has been made regarding the type of aging applied to the specimens: (**c**) salt spray aged and (**d**) immersion aged. Black dots represent the observed experimental data, the dashed line represents the ordinary least squares (OLS) fitted regression line, and the orange lines represent hundred posterior predictive regression lines calculated by taking regression parameters from the posterior distribution.

The mode I fatigue crack growth rate curves of the aged and unaged specimens are plotted and shown in Figure 6. With respect to the aged specimens, an additional distinction is made as to the type of aging procedure applied to the specimens, and their corresponding plots are shown in Figure 6c,d. The data points exhibit significant scatter, which can be attributed to the presence of voids and discontinuities in the bondline observed during the post-mortem analysis of the fractured surface. It should be noted that the data points, and by extension the FCGR curve, are plotted until the crack is at the interface and the specimen is loaded under mode I condition. The data points after the crack deviates these conditions are omitted from analysis, i.e., once the crack starts to deviate from the interface towards the opposite interface. The results of the fatigue experiments and the influence of the aging procedure are discussed in detail. In addition to presenting the power law constants *C* and *m* for each condition, the similarity and/or difference in the slope of the FCGR curves are discussed in Section 3.3.

### 3.1. Unaged Specimens

A total of six unaged specimens are tested under mode I fatigue loading; the loading sequence and the number of cycles to failure are presented in Table 1. The average critical load ${\left(F_{\mathrm{crit}}\right)}_{\mathrm{avg}}$ obtained from quasi-static testing is 674 N [39]. Given the limited number of available specimens, the fatigue test is initiated at a low load to ensure sufficient data points are captured within the stable crack growth region. First, specimen UA-DCB-07 is tested with an initial loading block of $F_{\max}=30\%{\left(F_{\mathrm{crit}}\right)}_{\mathrm{avg}}$ for 1.0 × 10^6^ cycles during which no crack growth is observed. The load is subsequently increased to 40%, 45% and 50% of ${\left(F_{\mathrm{crit}}\right)}_{\mathrm{avg}}$. Crack initiation and steady-state growth are observed during the latter two load stages, leading to final failure after 3.39 × 10^6^ cycles. To expedite the testing process, both the 30% and 40% block loading levels are omitted for subsequent specimens. Specimen UA-DCB-04 is tested at an additional intermediate load level of 47.5%; however, this does not yield additional insights compared to directly increasing the load from 45% to 50% and is therefore excluded in further tests.

**Figure 7:**
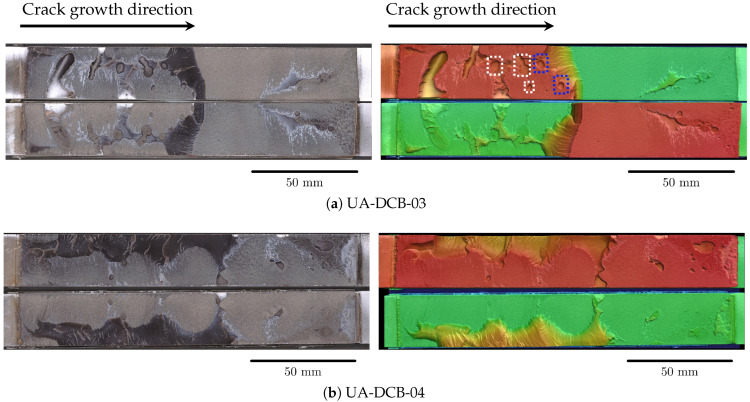

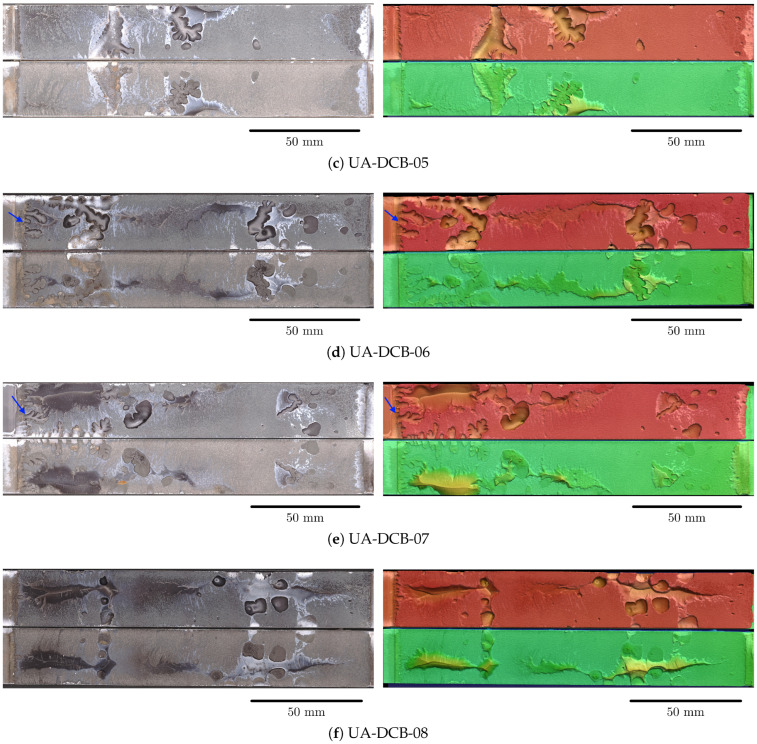
The optical fracture surface image and the corresponding 3D surface scan of the unaged DCB specimens is shown on the left and right, respectively. The colors in the surface scan represent the height of the features measured by the profilometer; red being closest to the reader and green being the farthest.

The FCG curve of the unaged DCB specimens is plotted in Figure 6a. As previously stated, the scatter in the data points can mainly be attributed to the presence of voids and discontinuities in the bondline which is ascertained from analyzing the fracture surfaces of the specimens. Figure 7 shows the fracture surfaces of unaged DCB specimens captured using the 3D optical profilometer (Keyence VR-5200); on the left, optical scans of both the DCB arms are shown, and on the right the corresponding 3D surface scan of the fracture surface is shown. The colors in the surface scan represent the height of the features measured by the profilometer; red being closest to the reader and green being the farthest. From the images, it is evident that the specimens fail predominantly due to adhesive failure, which is typical as the initial crack is located at the interface of the steel adherend. Additionally, regions of cohesive failures are also observed; however, these regions are smaller in comparison with the adhesive failure region. Interestingly, specimen UA-DCB-04 (Figure 7b) shows a large region of cohesive failure which is predominantly located at one face of the specimen, while the other face shows adhesive failure. This leads to a non-uniform crack length at the two faces of the specimen. Figure 5b shows the crack length measured at the front and back of the specimen as a function of the number of cycles. Also plotted is the crack length at the center of the specimen calculated using Equation (5), which shows that this approach provides a more accurate representation of the crack growth.

It is evident from Figure 7 that the specimens contain regions of adhesive discontinuities and voids which likely developed during the manufacturing and curing process. From Figure 7a,c,d,f, it is clear that the specimens UA-DCB-03, UA-DCB-05, UA-DCB-06, and UA-DCB-08 contain discontinuity in the adhesive layer along the entire width of the specimen. Additionally, specimens UA-DCB-06 and UA-DCB-07 contain a region of discontinuities at the initial crack location (indicated by blue arrows), which looks similar to a *Lichtenberg figure*, but without the sharp points. This leads to a reduced effective bonding area, as only ligaments of adhesive are bonded to the adherend. It is also interesting to note that only specimen UA-DCB-03 shows the transition of the crack front from one interface to the other. This transition phenomenon is discussed further in Section 3.2.

### 3.2. Aged Specimens

**Figure 8:**
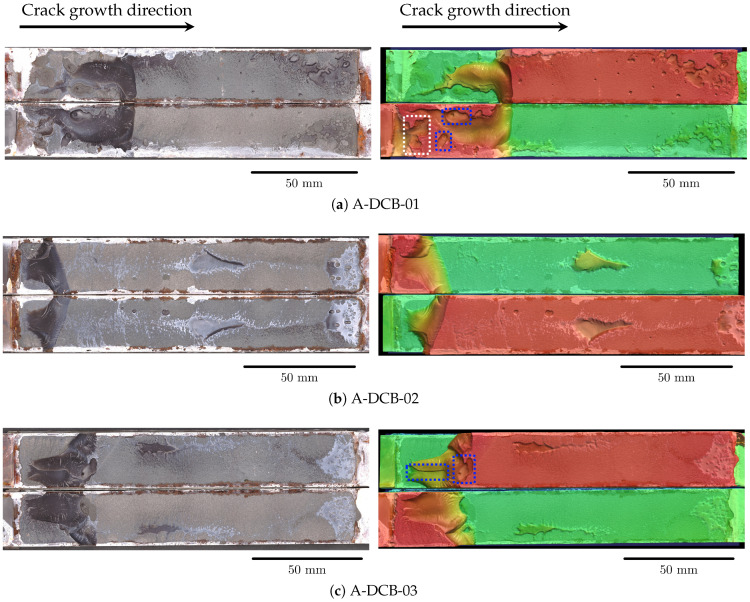

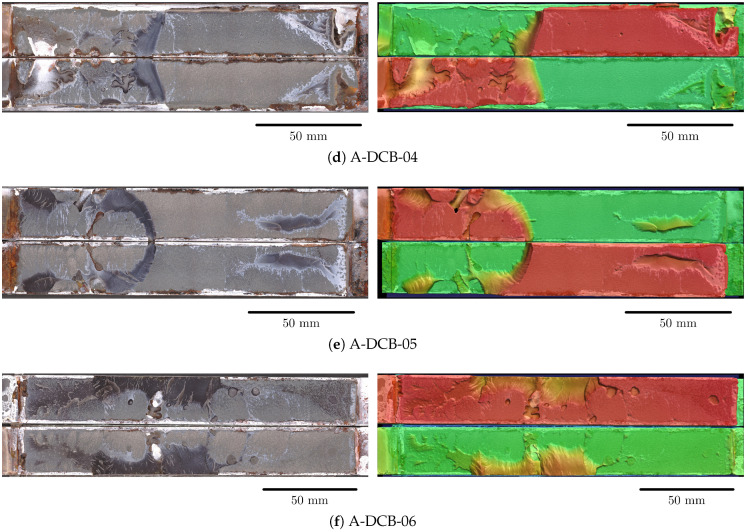
The optical fracture surface image and the corresponding 3D surface scan of the aged DCB specimens are shown on the left and right, respectively. The colors in the surface scan represent the height of the features measured by the profilometer, red being closest to the reader and green being the farthest.

A total of six aged specimens are tested under mode I fatigue loading. The loading sequence, number of cycles to failure, and the aging methodology are presented in Table 2, and the corresponding FCG curve is plotted in Figure 6b. The average critical load ${\left(F_{\mathrm{crit}}\right)}_{\mathrm{avg}}$ obtained from quasi-static testing is 567.4 N [39]. This average critical load is used as a base for both salt-spray-aged and immersion-aged specimens. The fracture surfaces of the aged DCB specimens captured with the optical profilometer are shown in Figure 8.

#### 3.2.1. Salt-Spray-Aged Specimens

Similar to the unaged specimens, the loading blocks for the salt-spray-aged specimens are increased after every 1 × 10^6^ cycles. Specimen A-DCB-01 is tested with an initial load block of $F_{\max}=45\%{\left(F_{\mathrm{crit}}\right)}_{\mathrm{avg}}$. This block-load level does not result in any crack growth after 1 × 10^6^ cycles. However, the next load levels of 50% and 55% do result in crack growth before eventual failure of the specimen. Accordingly, a load level of $50\%{\left(F_{\mathrm{crit}}\right)}_{\mathrm{avg}}$ is chosen as the initial load level for specimen A-DCB-03; however, at this load level, there is no crack growth observed. Hence, for the last salt-sprayed specimen A-DCB-06, the initial load level is set to $55\%{\left(F_{\mathrm{crit}}\right)}_{\mathrm{avg}}$, but this specimen fails after 0.88 × 10^6^ cycles within the first loading block. This shows a variability in the crack growth at a certain load level within the salt-spray-aged specimens. The FCG curve for the salt-spray-aged specimens is shown in Figure 6c.

**Figure 9:**
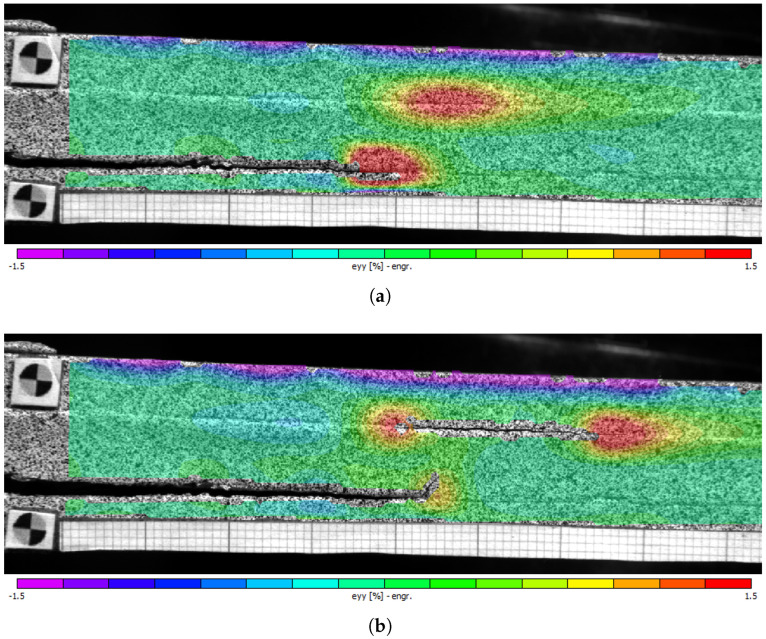
DIC result showing the peel strain distribution on the surface of the DCB specimen. (**a**) Development of peel strain at the opposite steel–adhesive interface and (**b**) showing the disbond at the interface and the deviation of the crack front from one interface towards the opposite interface.

From Figure 8, it is evident that the salt-spray-aged specimens fail due to a combination of adhesive and cohesive failure. However, it is noteworthy that, with the exception of specimen A-DCB-06 (Figure 8f), the crack front in the other two specimens (Figure 8a,c) transitions from the initial steel–adhesive interface to the opposite interface, similar to the unaged specimen UA-DCB-03. Examining the fracture surfaces, it is observed that this transition typically occurs near regions (highlighted in blue) containing voids. However, it is too simplistic to conclude that voids aid the transition of the crack front from one interface to the other. Curiously, in specimen UA-DCB-03 and A-DCB-01, voids are present at regions (highlighted in white) prior to where the crack-front begins to transition. In these regions, the failure mechanism resembles the cohesive failure observed in unaged specimens. The analysis of strain fields in the specimen, based on DIC images, reveals the development of peel strain at the opposite interface during the experiment, as shown in Figure 9a. As the experiment progresses, a disbond initiates at this location, gradually increasing in size, ultimately causing the original crack to deviate as illustrated in Figure 9b. The development of peel strain at the opposite interface may be attributed to either a weakened steel–adhesive interface caused by aging or the bending of the steel adherends as reported by [40]. However, in the present research, the applied loads are comparatively lower, and post-mortem observations do not indicate any permanent plastic deformations in the steel adherends. Nevertheless, it is plausible that a combination of bending and a weakened interface contribute to the disbond at the opposite steel–adherend interface.

#### 3.2.2. Immersion-Aged Specimens

**Figure 10:**
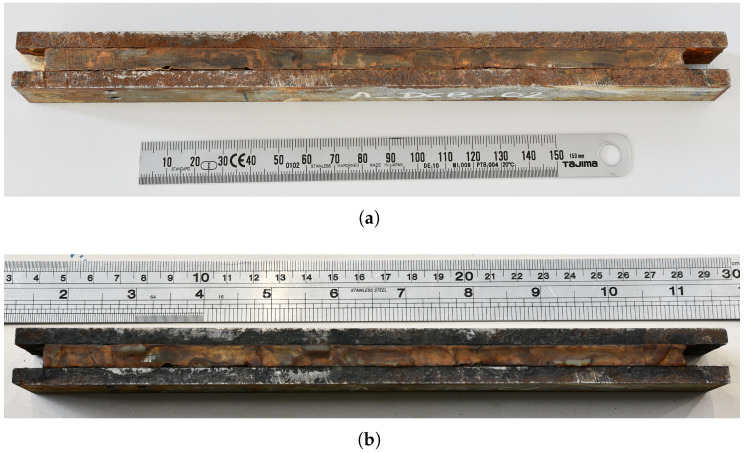
Photographs of the DCB specimens (**a**) before and (**b**) after immersion into the salt water bath. The rust from the specimen is removed by lightly sanding the surface before being photographed, hence the shiny regions in the steel adherend.

The immersed DCB specimens are removed from the water bath and blotted with paper towels to remove the excess water before being painted white on both faces to aid crack length measurement. Care is taken to ensure that the specimen is not completely dry before testing; this is to observe if any moisture ingresses into the specimen along the crack path as the fatigue experiment progresses. This also means that no DIC speckles could be applied to this set of specimens. Figure 10 shows photographs of the DCB specimens before and after immersion into the salt water bath. The rust from the specimens is removed by lightly sanding the surface before being photographed; this leads to the shiny regions seen in the steel adherend. The loading blocks for the immersion-aged specimens are decided to be increased after every 1 × 10^6^ cycles, similar to the unaged and salt-spray-aged specimens. However, this leads to the moisture being dried out of the specimen. Hence, after testing the specimen A-DCB-02 for 2 × 10^6^ cycles, it is decided to increase the load after every 1 × 10^5^ cycles, to accelerate the testing. The FCG curve for the immersion-aged specimens is shown in Figure 6d, which exhibits a larger variability in data points when compared to the unaged and salt-spray-aged specimens. Figure 10 shows the photographs of specimens before and after immersion in the salt-water bath. Visual inspection of the specimen after immersion shows localized discoloration and swelling, which is likely due to the moisture ingress in the adhesive. Interestingly enough, see Figure 8, the immersed specimen shows more traces of corrosive byproducts at the edges of the specimen and near the initial crack-tip, but no signs of corrosive byproducts are visible in the bonded region. This illustrates that the immersion of the joint does not lead to corrosion or disbond in the bonded region resulting from moisture ingress.

Analyzing the fracture surfaces (Figure 8b,d,e), it is observed that the region of cohesive failure is significantly less in comparison to the cohesive failure region of unaged and salt-spray-aged specimens. Also noteworthy is the transition of the crack from one interface to the other over a shorter distance, suggesting that the crack travels *almost* vertically along the adhesive layer. This is in contrast to the salt-spray-aged specimens where the crack front transitions over a longer distance. Additionally, in the immersion-aged specimens, voids, even though present, do not influence the crack path transition from one interface to the other. Once the crack transitions to the opposite interface, the crack propagates rapidly and the specimen fails within the start of the next data collection cycle. This can be observed by the greyish-white regions seen in the optical scans of the fracture surfaces.

The crack length measurement employed in the current research (Section 2.5) assumes that the crack front is a straight line along the width between the two faces of the specimen. This in reality is not the case due to the presence of voids and discontinuity in the bonded area as seen in the fracture surfaces of the specimens under different aging conditions (Figure 7 and Figure 8). The assumption of a straight crack front is a best-case scenario since the presence of the crack front at the adhesive–steel interface does not allow it to be measured by conventional methods such as measuring fatigue striations, or beach marking [53,54].

### 3.3. Data Analysis

The data analysis in the current section is performed in software R (version 4.4.1) using packages ‘dplyr v1.1.4’, ‘readr v2.1.5’, ‘stats v4.4.1’, and ‘tidyverse v2.0.0’ [55,56,57,58]. A total of i=480 data points are obtained from experiments within the three categories, i.e., unaged, salt-spray aged, and immersion aged. The experimental data are fitted using the ordinary least squares (OLS) approach to obtain the values of parameters *C* and *m* of the power law Equation (1) and are presented in Table 3.

The same values are also obtained from the MMR model, in which $\beta_{0}$, $\left(\beta_{0}+\beta_{2}\right)$ and $\left(\beta_{0}+\beta_{4}\right)$ represent the intercepts and $\beta_{1}$, $\left(\beta_{1}+\beta_{3}\right)$ and $\left(\beta_{1}+\beta_{5}\right)$ the slopes of the regression lines for unaged, salt-spray-, and immersion aged, respectively. Additionally, the MMR model also tests for the differences in slope by performing ANCOVA on the interaction terms. For the unaged and salt-spray-aged specimens, the interaction term has a t-score of t=−1.728 with a corresponding *p*-value =0.0846>α; $H_{0}$ cannot be rejected, and it can be assumed that both unaged and salt-spray-aged specimens have consistent slopes. However, for the immersion-aged specimens, t=−6.033 with a corresponding *p*-value $=3.24\times 10^{-9}<\alpha$; and thus, $H_{0}$ can be rejected in favor of $H_{1}$, which implies that the slopes of the unaged specimens and immersion specimens are significantly different. In a similar vein, a statistical test is performed to understand the influence of the additional aging process of immersing the specimen in a saltwater bath, which yields a t-score t=−4.395 and corresponding *p*-value $=1.83\times 10^{-5}<\alpha$. From this it can be concluded that the slopes of salt-spray-aged and immersion-aged specimens are significantly different. The results of these statistical tests reinforce what is visually evident from the slopes of the OLS regression lines seen in Figure 6.

**Figure 11:**
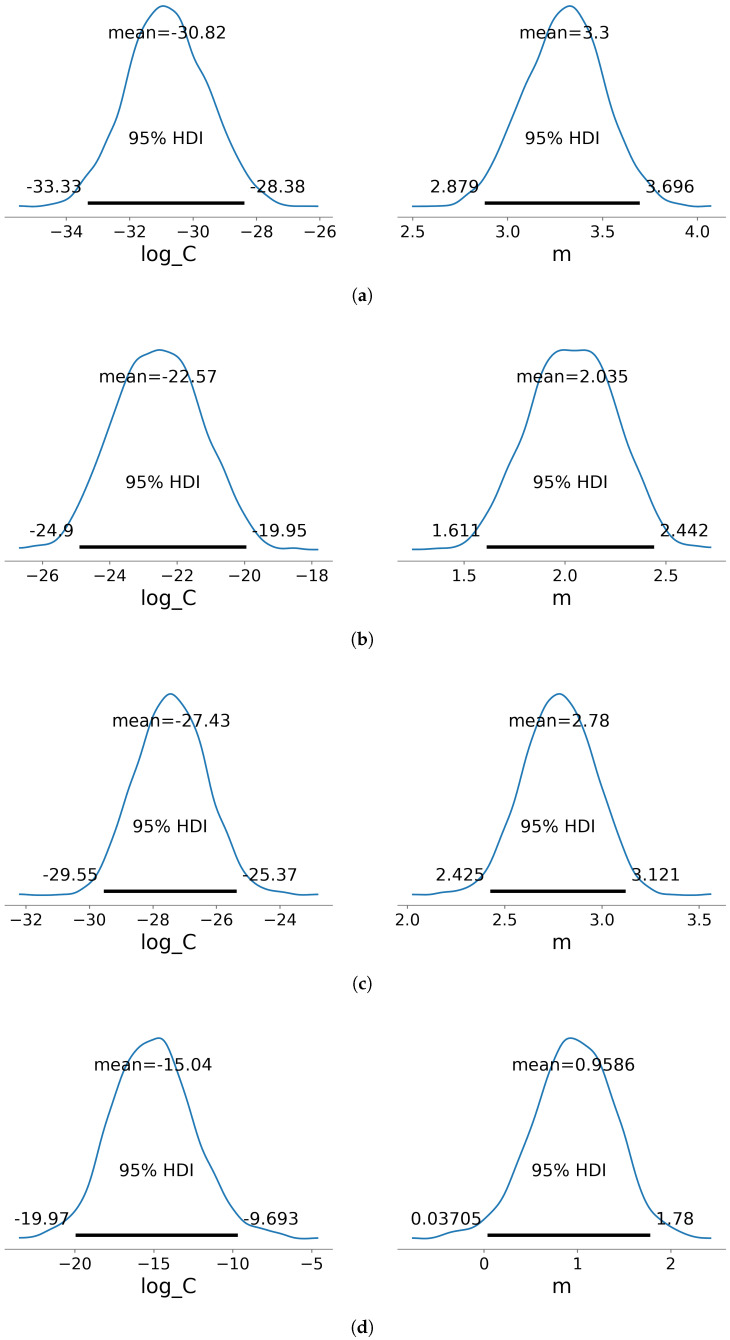
Posterior distribution of the linearized power law coefficients logC and *m* of the (**a**) unaged and (**b**) aged DCB specimens. The distribution is also plotted for the different aging methods applied to the specimens: (**c**) salt spray aged and (**d**) immersion aged. The plots indicate the mean value of this distribution along with the 95% credible interval.

Due to the scatter and limited number of data points gathered from experiments per group, it is reasonable to estimate the power law coefficients as a distribution of possible values rather than single-point estimates. To this end, Bayesian linear regression is implemented on the dataset using the No U-turn sampler (NUTS) in PyMC library v5.16.1 [59]. The posterior distributions of the linearized power law coefficients logC and *m* for different aging conditions of the specimens are shown in Figure 11. The plots also indicate the mean value of the distribution along with the 95% credible interval (CI) for the coefficients, indicating the range within which the coefficients are expected to lie with 95% probability given the observed data. These values are also summarized in Table 4. Additionally, a posterior predictive plot of regression lines is generated by sampling from the posterior distributions of logC and *m*. These lines, depicted in orange in Figure 6, illustrate multiple plausible “best-fit” regressions.

Observing the FCGR plots, it is evident that the crack growth occurs in the *G*_max_ range 100 J mm^−2^ to 1100 J mm^−2^. The immersion-aged specimen exhibits a shallower slope in comparison to the unaged and salt-spray-aged specimens. The crack growth rate is also a magnitude higher for the immersion-aged specimens at lower *G*_max_ values. Additionally, the lower bound 95% credible interval value of *m* for the immersion-aged specimens shows that there is a plausibility that the crack growth rate is independent of the strain energy release rate. This showcases a clear influence of immersion on the response of the specimen to fatigue loading.

A caveat to the results presented in this research has to be noted. Due to the small sample size of the unaged specimens and even smaller sample size for the salt-spray-aged and immersion-aged specimens, a definitive conclusion on the mechanical behavior of thick adhesive bondline DCB specimens cannot and should not be made. This research provides a preliminary insight into the fatigue crack growth behavior of DCB specimens, manufactured in shipyard conditions, under different environmental conditions. The values presented in this paper describe a general trend for various aging conditions, notwithstanding the scatter and small sample size; the values obtained using OLS and Bayesian linear regression would tends towards each other as the sample size tends towards infinity.

## 4. Conclusions

DCB specimens with thick adhesive bondline and initial crack at the steel–adhesive interface were manufactured under shipyard conditions and subjected to mode I cyclic loading to determine the fatigue crack growth rate. The influence of the aging methodology on the crack growth rate was also investigated by subjecting the specimens to salt-spray and immersion aging. A dual-camera system was used to monitor the crack growth in the specimen, and the Kanninen–Penado model was used to determine the energy release rate and the fatigue crack growth rate. All specimens exhibited scatter in the data which can be attributed to the presence of voids and discontinuities in the bondline. The power law coefficients logC and *m* were determined using ordinary least square regression and Bayesian linear regression.

The results showed that the slopes of the unaged and salt-spray-aged specimens are consistent, however, the slopes of the unaged and immersion-aged specimens are significantly different. Bayesian linear regression additionally helped determine the posterior distribution of the power law coefficients to illustrate the plausible values and slopes for each aging condition. The result show that for all conditions, the crack growth occurs in the *G*_max_ range 100 J mm^−2^ to 1100 J mm^−2^ and is a magnitude higher for the immersion-aged specimens at lower *G*_max_ values, showing a clear influence of immersion aging on the crack growth rate.

Further research with specimens manufactured under laboratory conditions and a larger number of specimens are necessary to generalize the behavior of DCB specimens with thick adhesive bondlines. On balance, the results highlights the impact of aging on the fatigue property, underscoring the importance of considering environmental effects in the qualification of such joints for marine applications.

## Figures and Tables

**Table 1:** Summary of the mode I fatigue test results for unaged specimens.

Specimen	Block Loading Sequence	Number of Cycles to Failure
UA-DCB-03	45%	783,211
UA-DCB-04	45%, 47.5%, 50%	2,042,765
UA-DCB-05	45%, 50%, 55%	2,137,778
UA-DCB-06	45%, 50%	1,010,026
UA-DCB-07	30%, 40%, 45%, 50%	3,390,261
UA-DCB-08	45%, 50%	1,430,000

**Table 2:** Summary of the mode I fatigue test results for aged specimens.

Specimen	Block Loading Sequence	Number of Cycles to Failure	Aging Methodology
A-DCB-01	45%, 50%, 55%	2,498,006	Salt-spray
A-DCB-02	45%, 50%, 55%, 60%, 65%, 70%	2,383,010	Salt-spray + Immersion
A-DCB-03	50%, 55%, 60%	2,917,432	Salt-spray
A-DCB-04	50%, 55%, 60%	165,024	Salt-spray + Immersion
A-DCB-05	45%, 50%, 55%, 60%, 65%, 70%, 80%	631,651	Salt-spray + Immersion
A-DCB-06	55%	878,172	Salt-spray

**Table 3:** The power law coefficients calculated using OLS for unaged and aged data.

Condition	C	m
Unaged specimens	2.3726 × 10^−14^	3.3911
Aged specimens (combined)	1.0907 × 10^−10^	2.0978
- Salt-spray-aged specimens	8.8870 × 10^−13^	2.8334
- Immersion-aged specimens	9.2916 × 10^−8^	1.1497

**Table 4:** The mean, standard distribution (SD), and 95% credible interval (CI) of the posterior distribution of the linearized power law coefficients logC and *m* for different aging conditions.

Specimen Condition	Coefficient	Mean	SD	2.5% CI	97.5% CI
Unaged	logC	−30.858	1.214	−33.072	−28.284
	*m*	3.307	0.201	2.897	3.690
Aged (combined)	logC	−22.572	1.259	−25.100	−20.150
	*m*	2.036	0.210	1.630	2.453
- Salt spray	logC	−27.425	1.115	−29.670	−25.205
	*m*	2.780	0.186	2.383	3.127
- Immersion	logC	−15.289	2.493	−20.070	−10.467
	*m*	1.000	0.419	0.197	1.805

## Data Availability

The raw data supporting the conclusions of this article will be made available by the authors on request.
